# Mast Cell Leukemia: An Update with a Practical Review

**DOI:** 10.3390/cancers15061664

**Published:** 2023-03-08

**Authors:** Magda Zanelli, Martina Quintini, Salvatore Magnasco, Lara Aprile, Andrea Palicelli, Maurizio Zizzo, Francesca Sanguedolce, Stefano Ricci, Saverio Pancetti, Valeria Zuccalà, Veronica Martino, Giuseppe Broggi, Rosario Caltabiano, Alberto Cavazza, Paola Parente, Cristina Mecucci, Giovanni Martino, Stefano Ascani

**Affiliations:** 1 Pathology Unit, Azienda USL-IRCCS di Reggio Emilia, 42123 Reggio Emilia, Italy; 2 Hematology, Centro di Ricerca Emato-Oncologica–C.R.E.O., University of Perugia, 06129 Perugia, Italy; 3 Pathology Unit, Ospedale SS Annunziata di Taranto, 74121 Taranto, Italy; 4 Hematology Unit, Presidio Ospedaliero S.G. Moscati di Taranto, 74010 Taranto, Italy; 5 Surgical Oncology Unit, Azienda USL-IRCCS di Reggio Emilia, 42123 Reggio Emilia, Italy; 6 Pathology Unit, Policlinico Riuniti, University of Foggia, 71122 Foggia, Italy; 7 Pathology Unit, Humanitas University, Pieve Emanuele, 20072 Milano, Italy; 8 Pathology Unit, Humanitas Research Hospital-IRCCS, Rozzano, 20089 Milano, Italy; 9 Pathology Unit, Pugliese-Ciaccio Hospital Catanzaro, 88100 Catanzaro, Italy; 10 Department of Medical and Surgical Sciences and Advanced Technologies “G.F. Ingrassia” Anatomic Pathology, University of Catania, 95123 Catania, Italy; 11 Pathology Unit, Fondazione IRCCS Casa Sollievo della Sofferenza, San Giovanni Rotondo, 71013 Foggia, Italy; 12 Pathology Unit, Azienda Ospedaliera Santa Maria di Terni, University of Perugia, 05100 Terni, Italy

**Keywords:** mast cell leukemia, systemic mastocytosis, MCL-AHN, MCL-AMN, KIT mutation, midostaurin

## Abstract

**Simple Summary:**

Mast cell leukemia (MCL) is a rare and aggressive subtype of systemic mastocytosis (SM). MCL diagnosis requires fulfillment of diagnostic criteria for SM plus at least 20% immature/atypical mast cells on bone marrow smear. Based on different clinicopathological characteristics, MCL is subdivided into the following variants: primary (de novo) or secondary (from a prior SM form); acute with C-findings (where C-findings represent organ damage requiring cytoreductive treatment) or chronic without C-findings; and finally, MCL with or without an associated hematological neoplasm. The latter category was renamed MCL with an associated myeloid neoplasm in the 2022 International Consensus Classification (ICC). The relevance of the distinction between the leukemic and aleukemic forms, based on the percentage of circulating mast cells, is under discussion. In this study, we review the current knowledge on MCL with the aim of increasing clinicians’ and pathologists’ awareness of this rare, still incompletely understood disease with a grim prognosis.

**Abstract:**

Mast cell leukemia (MCL) is the leukemic form of SM with at least 20% mostly immature mast cells on bone marrow aspirate. MCL may develop de novo, in the absence of a prior SM, or it may represent a progression from a previous SM. MCL may be sub-divided into the more frequent, aggressive acute form with signs of organ damage (C-findings) and the chronic form lacking C-findings and presenting a more stable course, although over time, progression to acute MCL is common. The 2022 WHO subtype of MCL with an associated hematological neoplasm was renamed MCL with an associated myeloid neoplasm in the 2022 International Consensus Classification (ICC). The relevance of the distinction between the leukemic and aleukemic forms based on the percentage of circulating mast cells is a matter of debate. The current knowledge on MCL is restricted mainly to single reports or case series with a limited number of larger studies. Our aim is to provide a comprehensive overview of this rare disease in terms of clinical manifestations, morphology, phenotype, molecular characteristics, differential diagnosis, outcome and treatment. A general overview on mastocytosis is also included.

## 1. Introduction

Mast cell leukemia (MCL), which was first described by Joachim in 1906, represents the most uncommon form of systemic mastocytosis (SM) and is seen in less than 5% of all SM patients [1,2,3,4,5,6,7,8,9]. 

It is characterized by the leukemic expansion of mostly immature mast cells (MCs) in the bone marrow (BM) and other organs, with organ damage and a poor prognosis in the majority of cases with a median overall survival (OS) of 1.6 years [2,3,4,5,6,7,8,9]. 

The diagnosis of MCL requires the presence of the diagnostic criteria for SM plus at least 20% atypical, immature MCs in BM smears. In the case of suboptimal BM smears, according to the 2022 International Consensus Classification (ICC), MCL diagnosis can be performed on BM biopsy, which is usually diffusely infiltrated by atypical/immature MCs (Table 1) [10].

Under discussion is the real importance of sub-dividing MCL into the leukemic variant with at least 10% atypical/immature MCs in the peripheral blood (PB) and the aleukemic variant with less than 10% circulating MCs [10]. A recent report on the largest cohort of MCL patients has interestingly noted that the presence of any circulating MCs even below the 10% threshold may negatively impact prognosis [9].

MCL is a rather heterogeneous disease that is sub-divided into an acute form and a chronic form on the basis of the presence or not of organ damage (C-findings). The acute form, occurring in the majority of patients (60–90%), has a rapid development and an aggressive course with massive organ damage; whereas the chronic form has a more prolonged course without rapid organ damage, although, over a variable length of time, progression to acute MCL seems to be the rule [11].

In addition, the disease may develop de novo (so-called primary MCL) or it may be secondary when an antecedent MC neoplasm is present; finally, MCL may occur alone or with an associated hematological neoplasm, the so-called MCL-AHN as per the 2022 WHO classification [3]. This category has been renamed MCL with an associated myeloid neoplasm (MCL-AMN) in the 2022 ICC because hematologic neoplasms associated with SM or MCL are predominantly myeloid neoplasms often clonally related with SM [10]. MCL variants are summarized in Table 2.

Due to the rarity of MCL, both the pathogenesis and treatment of the disease are not well defined.

Herein, we aim to highlight the clinicopathologic features of this aggressive and still incompletely known disease.

## 2. Overview on Mastocytosis, SM Diagnostic Criteria and Different Subtypes of SM

Mastocytosis is a very heterogeneous group of neoplastic MC disorders, ranging from forms with an indolent clinical behavior such that patients may have an almost normal life-expectancy to other forms with an aggressive course and short survival due to multi-organ involvement [12,13,14,15,16,17].

The 2022 WHO classification continues to recognize three main forms of mastocytosis [3]:

- cutaneous mastocytosis (CM) with skin-limited disease, which is more common in children and which presents good biological behavior;
- SM with involvement of one or more extra-cutaneous organs, which is more frequent in adults and has a less favorable outcome;
- mast cell sarcoma (MCS), a very uncommon, localized and high-grade MC neoplasm [2,3,14,15,18,19,20,21,22].

According to the 2022 WHO classification, the diagnosis of SM requires that the major criterion plus one minor criterion or at least three minor criteria are fulfilled [2,3], whereas in the 2022 ICC, SM is defined by the presence of the major criterion or, in its absence, by the presence of at least three minor criteria [10].

The minor criteria have been slightly refined with the introduction of CD30 expression and the presence of any *KIT* mutation as minor diagnostic criteria [3,10].

- The major criterion consists of multifocal, dense aggregates, each of at least 15 MCs, in BM biopsy and/or another extra-cutaneous organ.

Because the MC aggregates may not be always easily identified in routinely stained sections, the 2022 ICC has added in the definition of the major criterion the confirmation of tryptase or CD117 expression [10]. The diagnostic criteria for SM mastocytosis are summarized in Table 3.

SM is further classified into different subgroups based on whether or not B- and/or C-findings are present. B-findings are signs of an excessive MC burden in the tissues, whereas C-findings are signs of specific MC-related organ damage (where C stands for requiring cytoreductive therapy).

- The following features are recognized as B-findings: >30% of BM cellularity consisting of MCs and serum total tryptase >200 ng/mL; dysplasia or myeloproliferative features in non-MC lineages that are not enough for the diagnosis of other myeloid neoplasms with normal or only slightly abnormal blood counts; hepatomegaly without impairment of liver function; splenomegaly without hypersplenism and/or abnormal size of lymph nodes.

In the current 2022 WHO classification, the presence of an NM-000222:KIT p.D816V mutation with variant allele frequency (VAF) ≥ 10% in BM cells or PB leukocytes is considered a B-finding [3].

- The following features qualify as C-findings: one or more cytopenia due to diffuse BM infiltration; hepatomegaly with damaged liver function; splenomegaly with hypersplenism; malabsorption and weight loss due to gastrointestinal (GI) MC infiltration; and osteolytic bone lesions.

Based upon B- and C-findings, SM is sub-divided into indolent SM (ISM; with or without one B-finding and no C-findings), smoldering SM (SSM; at least two B-findings, but no C-findings) and ASM (at least one C-finding).

The current WHO 2022 classification recognizes bone marrow mastocytosis (BMM) as a separate subtype of SM characterized by a lack of skin lesions and B-findings and a basal serum tryptase level below 125 ng/mL [3]. In the 2022 ICC, BMM is recognized as a clinico-pathologic variant of ISM [10].

In addition, the current 2022 WHO classification considers well-differentiated systemic mastocytosis (WDSM) as a morphological pattern which can be present in any subtype of SM. WDSM is characterized by the presence of round and granulated MCs diffusely infiltrating BM. WDSM usually lacks *KIT 816* mutation, and MCs are often negative for CD2 and CD25 and positive for CD30 [3].

Non-advanced SM subtypes are the most frequent and include BMM, ISM and SSM. Advanced SM includes ASM, SM-AHN (SM-AMN) and MCL [3,9,10,23,24].

## 3. MCL: Defining Criteria, Leukemic and Aleukemic Variants, Primary and Secondary Variants

Because of the rarity of MCL, accounting for 1% of adult SM, our current knowledge of the disease, including its clinicopathologic features, molecular characteristics, prognosis and treatment, is mainly restricted to case reports and only limited case series [6,25,26,27].

To date, only four studies have systematically reported MCL.

In 2013, Georgin-Lavialle et al. reviewed clinico-pathologic data of 51 published MCL cases [4]; in 2017, Jawhar et al. reviewed 28 cases of MCL diagnosed between 2008 and 2016 in Germany [5]; in 2017, Jain et al. presented a single American center experience with 218 SM cases, among which 13 MCL were present [6]; more recently in 2022, Kennedy et al. evaluated the largest cohort of MCL patients (92 cases) from the European Competence Network on Mastocytosis (ECNM) registry [9].

MCL is the leukemic variant of SM, and consequently, for MCL diagnosis, criteria of SM are required.

Once SM is diagnosed, the next step to establish a MCL diagnosis is to identify the presence of at least 20% immature/atypical MCs in BM smear [2,3].

Although the threshold of 20% MCs is generally based upon the evaluation of BM smears, according to the 2022 ICC, MCL diagnosis can be performed even on BM biopsy in case of suboptimal aspirate (dry tap) [10].

In MCL, MCs may be detected in PB, and based on the number of circulating MCs, MCL has been sub-divided into the leukemic variant with at least 10% circulating MCs and the more frequent aleukemic variant (less than 10% of circulating MCs) that accounts for 60–90% of MCL cases [3].

In the study by Kennedy et al., 12.4% of patients had circulating MCs, but the criteria for leukemic MCL (10% circulating MCs) were met only in 4.5% of patients [9].

Compared to previous studies, in the report by Kennedy et al., the presence of any circulating MCs was found to negatively impact prognosis, reducing OS, and, therefore, the observation that any number of circulating MCs is associated with inferior outcomes should prompt reconsideration of the current threshold of 10% MCs to define leukemic MCL [9].

In the 2022 ICC, it is recommended to report the presence of circulating MCs due to its prognostic significance; however, the percentage of circulating MCs does not justify the distinction between the aleukemic and leukemic forms [10].

MCL may occur de novo (primary MCL) or secondary to a prior MC neoplasm (Table 3).

In general, in SM patients, the risk of transformation is rather low. In a large cohort of 342 SM patients, Lim et al. reported a 6% risk of transformation and, in the majority of cases (86%), SM transform to AML, with only 13% to MCL [21].

Secondary MCL commonly arises from SM-AMN (83–86%) and less often from ASM, while progression from ISM is very rare [10].

In the series of 28 MCL cases reported by Jahwar, about one third of patients had secondary MCL (sMCL), and in all cases, sMCL progressed from advanced SM, while no direct progression from ISM to MCL was observed, confirming that progression to MCL usually occurs mainly from SM-AHN or ASM [5].

Unlike in the study by Jahwar et al., in the recent cohort by Kennedy et al. among patients with sMCL, the antecedent MC disease was not only SM-AHN (52%) and ASM (26%) but even ISM (22%).

The median time to progression from initial diagnosis was 1.8 years, and progression to MCL was faster in patients with SM-AHN (0.7 years) than in patients with ASM (3.9 years) and in patients with ISM (5.3 years) [9].

## 4. MCL: Epidemiology and Clinical Manifestations

Advanced SM includes SM-AHN (40%), which is the second most common SM subgroup after ISM (46%); ASM represents approximately 12% of SM and MCL, which is the rarest form of SM, represents only 1% of SM [2,3].

According to the study by Georgin-Lavialle, the median age of MCL patients at diagnosis was 52 years (range 5–76 years) with a female predominance (F:M = 1.5:1). No familial cases have been described so far [4]. In the large MCL study by Kennedy et al., the median age at presentation was slightly older at 60.4 years (range 25.4–90.8 years) [9].

Primary MCL was prevalent (70.7%), whereas sMCL developed in 29.3% of cases [9].

MCL without AHN occurred in 66.3% of patients, whereas only 33.7% had AHN-MCL [9].

The clinical presentation of MCL is highly variable, and the different clinical manifestations depend on the leukemic involvement of multiple organs such as BM, bone, GI tract, liver, spleen and peritoneum.

Patients frequently have constitutional symptoms (fever, asthenia, night sweats and weight loss) and hepato-splenomegaly often associated with C-findings such as cytopenia, malabsorption, diarrhea, hepatic dysfunction, hypersplenism and osteolytic lesions.

Skin lesions are often absent in MCL, although more frequent cutaneous manifestations are reported in sMCL, certainly because of the antecedent mastocytosis [4].

Among GI manifestations, gastroduodenal ulcers are also reported [4].

Blood examination may reveal a variable percentage of circulating MCs. The aleukemic MCL (less than 10% circulating MCs) is the most frequent variant, accounting for 62% of all cases, 70% of sMCL cases and 55% of primary MCL cases [4].

The BM is always affected in MCL, resulting in peripheral cytopenia. Anemia and thrombocytopenia are frequent, and in primary MCL, the hemoglobin value is usually lower (9 g/dL) than in sMCL (11 g/dL); the PLT count is also usually lower in the primary form (82 G/L) than in sMCL (111 G/L) [4].

Serum tryptase is generally increased, with a value of over 200 μg/L in the majority of patients [9].

## 5. MCL: Acute Variant and Chronic Variant

As previously mentioned, the majority of MCL patients (60–90%) have signs and symptoms of organ damage (C-findings); thus, they are referred to as acute MCL patients [9,28].

In the largest study on MCL by Kennedy et al., most patients (86%) had acute MCL with at least one C-finding present; the majority of patients (68%) had both hematologic and non-hematologic C-findings; 25% had non-hematologic C-findings only; and 6% of patients had hematologic C-findings solely [9].

In the study by Kennedy et al., chronic MCL (lacking C-findings) was confirmed to be rare, comprising 14% of cases [9].

When patients with chronic MCL develop C-findings, the diagnosis of acute MCL has to be made [9,28].

As better detailed in the next paragraph on morphology, in most MCL cases, the majority of MCs have an immature morphology; however, in the chronic variant, MCs usually show a more mature morphology with round- or spindle-shaped cells.

It has to be mentioned that immature MCs with bi- or multi-lobed nuclei may be present even in the chronic form, whereas the presence of the most immature cells such as metachromatic blasts renders the diagnosis of chronic MCL very unlikely.

On the other hand, mature MCs with round- or spindle-shaped morphology are very uncommon in acute MCL.

Chronic MCL shows an immunophenotypic profile similar to that of the more common acute form, including the expression of tryptase, CD117, CD2, CD25, CD52 and CD30 [11,20,28]. However, some cases of chronic MCL occur in patients with WDSM and are characteristically negative for CD2 and CD25 [28].

From the molecular point of view, chronic MCL may harbor a *KIT D816V* mutation or rare mutations found also in WDSM. It has been supposed that chronic MCL may develop from an antecedent and long-lasting ISM with onset in childhood. Chronic MCL would develop when additional genetic alterations occur [20,28,29].

However, unlike patients with WDSM, who often present skin lesions, in chronic MCL, skin lesions are absent.

In spite of that, regressions of cutaneous lesions before BM involvement are described in WDSM, supporting the hypothesis that chronic MCL might have an antecedent phase characterized by skin involvement [10,28,30].

The clinical course of patients with chronic MCL is rather variable, as some patients develop C-findings and progress to acute MCL in a short time, whereas other patients have a more indolent and stable course for months or years, although progression to acute MCL seems to be the rule. Patients with the chronic form of MCL may respond to *KIT*-targeting therapies [28].

## 6. MCL: Morphology of MCs

As already mentioned, all patients with MCL need to fulfil SM diagnostic criteria and, therefore, the diagnostic approach starts from BM examination, which allows for the identification of not only MC aggregates but also a second hematologic disease if present.

**Figure 1:**
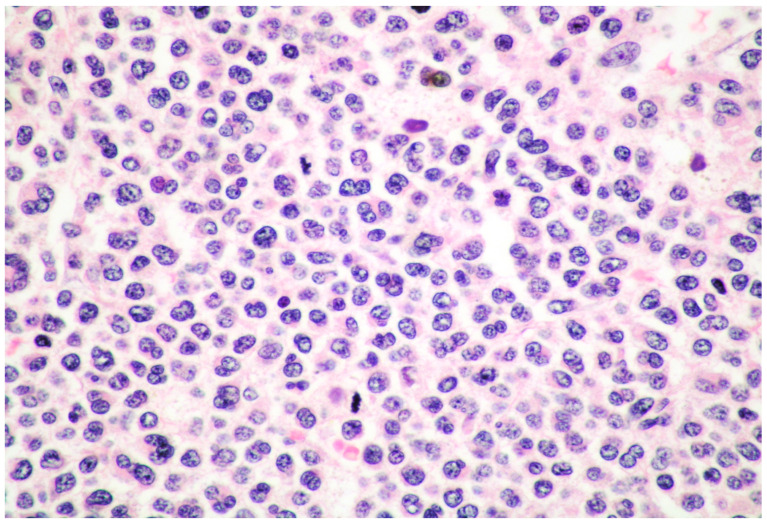
High power view of BM biopsy diffusely infiltrated by promastocytes (hematoxylin and eosin, magnification 300×; original image from Prof. S.A.).

Unlike in ISM, in MCL, BM infiltration is usually diffuse with a MC infiltrate ranging from 20% to 100%, completely replacing normal hematopoietic tissue (Figure 1) [2,3,4,5,6,7,8,9,10].

In SM, different morphological subtypes of MCs may be found, and they often vary according to the aggressiveness of the disease.

Atypical MCs may be divided into atypical mature MCs and atypical immature MCs.

Atypical mature MCs include two types of cells:

- spindle-shaped MCs characterized by an oval nucleus, elongated cytoplasmic processes and hypo-granulated cytoplasm, often with focal granule accumulation (atypical MC type I);
- well-differentiated MCs characterized by a round-shaped morphology, with a round nucleus and granulated cytoplasm.

**Figure 2:**
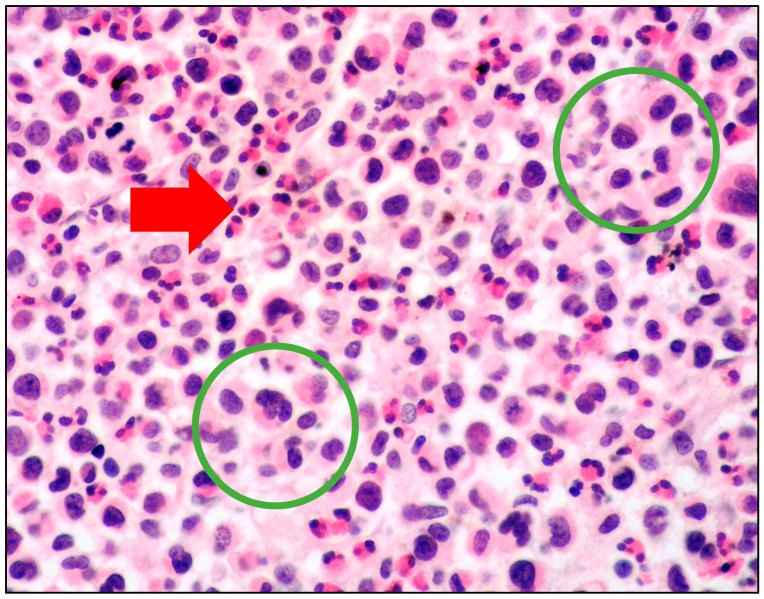
High-power view of BM biopsy diffusely infiltrated by atypical cells with pleomorphic nuclei (within green circles); in the background, numerous eosinophils were found (red arrow) (hematoxylin and eosin, magnification 400×; original image from Prof. S.A.

Atypical immature MCs include three types of cells:

- promastocytes which have bi-lobated or multi-lobated nuclei (Figure 2);
- metachromatic, granulated blasts;
- pleomorphic, multinucleated MCs.

Atypical mature MCs are more common in the more indolent forms of mastocytosis, whereas atypical immature MCs are more common in aggressive forms, including MCL [8,11,28,31,32]. Immature MCs may be easily unrecognized without the use of immunohistochemical stains for tryptase and CD117.

As mentioned in the dedicated paragraph, in the chronic variant of MCL, malignant MCs may sometimes show the same features encountered in ISM with a mature- and spindle-shaped morphology [8,11,28,31].

## 7. MCL: Immunophenotyping

MC aggregates in BM may not always be easily identifiable by standard stains (hematoxylin and eosin or Giemsa), in particular when MCs show an immature morphology and are hypo-granulated or in cases with an associated hematological malignancy such as AML overwhelming MC infiltrate [19].

**Figure 3:**
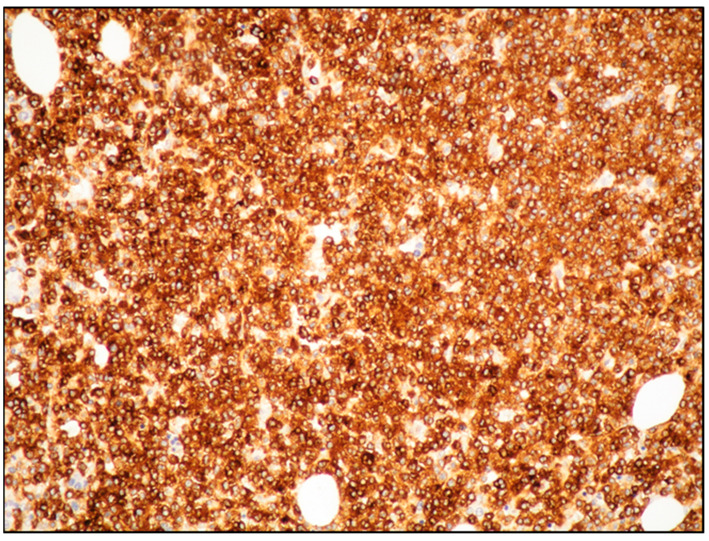
Medium power view of BM infiltrate highlighted by diffuse and intense CD117 positivity (CD117 immunostaining, magnification 100×; original image from Prof. S.A.).

**Figure 4:**
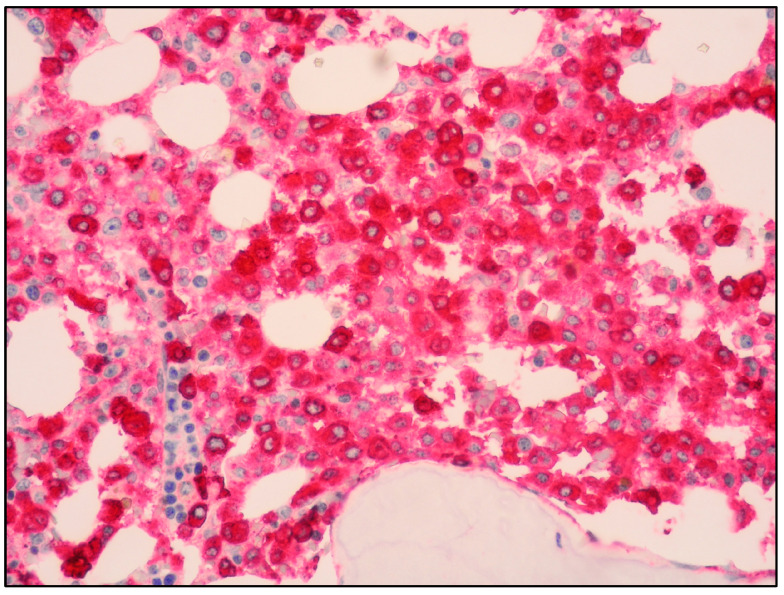
High-power view of BM infiltrate with diffuse and intense tryptase positivity (Tryptase immunostaining, magnification 400×; original image from Prof. S.A.).

The use of tryptase and CD117 generally allows the detection of MC infiltrates, even if small or with an immature morphology (Figure 3 and Figure 4) [19].

However, tryptase and CD117 expression alone is not indicative of the neoplastic nature of MCs.

**Figure 5:**
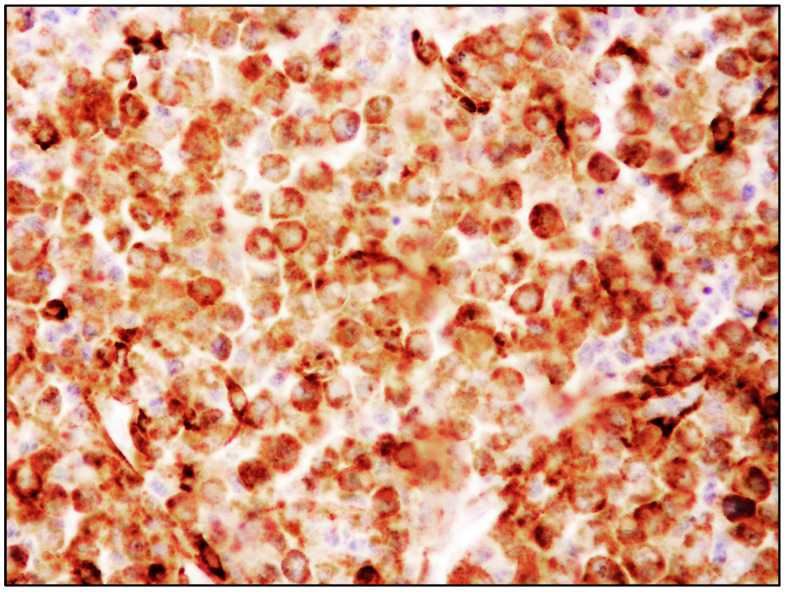
High-power view of BM infiltrate showing a diffuse and intense CD25 positivity (CD25 immunostaining, magnification 400×; original image from Prof. S.A.).

The immunohistochemical expression of CD25 and/or CD2 is currently recognized as a minor diagnostic criterion of SM, as the expression of these markers is indicative of the clonal origin of MC infiltrates (Figure 5) [2].

These two antigens are not expressed on normal MCs, myeloid precursors or immature MCs present in other myeloid neoplasms, including myelomastocytic leukemia (MML) [11].

CD25 is considered a more sensitive marker compared to CD2, which is not found in all cases of SM and decreases with disease progression [19].

In MCL, the phenotype of MCs is similar to that of SM, with MCs usually expressing tryptase, CD117, CD25, CD2, CD33, CD44 and CD9.

However, there are some differences between ISM and MCL. Compared with ISM, the percentage of cases positive for tryptase and CD2 is lower in MCL, as the expression of both tryptase and CD2 decreases with malignant progression [19].

The expression of CD2 is not detected in 48% of MCL, whereas CD25 expression is absent in 25% of MCL, and one third of MCL is reported to be negative for both CD25 and CD2.

In *KIT D816V*-positive MCL, the co-expression of CD25 and CD2 is more frequently found compared with *KIT D816V*-negative MCL (66% versus 25%).

CD52, HLA-DR, CD123 and CD30 are more often expressed in MCL compared with ISM.

In the current 2022 WHO classification and in the 2022 ICC, CD30 expression is recognized as a minor diagnostic criterion of SM [3,10]. The preferential expression of CD30 in neoplastic MCs of ASM and MCL suggests that CD30 expression may be caused by additional *KIT*-independent oncogenic events occurring when the disease progresses [33].

## 8. MCL: Molecular Features

The canonical *KIT D816V* mutation is found in over 80% of SM, but many other *KIT* mutations have been discovered so far [3,10].

The frequency of *KIT D816V* mutation is lower in MCL compared with ISM (>90% in ISM; 60–70% in MCL).

However, according to Jahwar et al., *KIT D816* mutations are more frequent than previously reported, as alternative mutations of *KIT* at position 816 such as *D816H* or *D816Y* may be found in a proportion (20%) of *KITD816V*-negative patients, leading to an overall incidence of *KIT D816* mutations in about 90% of MCL patients [5].

Therefore, *KIT D816V*-negative patients should be tested for other *KIT* mutations.

In particular, as some cases associated with noncanonical *KIT* mutations may be responsive to imatinib, complete gene sequencing of the *KIT* gene is suggested in cases without a *D816V KIT* mutation [10].

In the recent large MCL study by Kennedy et al., *KIT D816V* was detected in 73% of patients; alternative *KIT* mutations were found in 11% of patients; and no *KIT* mutations were found in 17% of patients [9].

Moreover, unlike the indolent forms of SM such as ISM and SSM, which are commonly uni-mutated malignancies with solely a *KIT* mutation, in most cases, advanced SM represents a multi-mutated disease usually presenting additional non-*KIT* mutations.

In addition to *KIT*, MCL may have a variety of myeloid-associated gene mutations such as *TET2*, *SRSF2*, *ASXL1*, *SF3B1* and *RUNX1* [4,5,6,9,10].

Mutations in the *SRSF2*, *ASXL1*, *RUNX1* (*S/A/R*) gene panel are observed in nearly 50% of MCL, and *S/A/R* mutations represent a significant adverse prognostic parameter [5,9,32]. Jahwar et al. showed that *S/A/R*-positive patients with MCL had a poor survival with resistance to different therapies compared to *S/A/R*-negative MCL patients [5,34].

In addition, these genetic alterations have been found to be more common in patients with MCL-AHN than in patients with MCL alone, and according to Kennedy et al., the presence of additional myeloid-associated mutations should alert clinicians and pathologists to evaluate the patient for a coexisting hematological malignancy [9].

## 9. SM-AHN (or SM/AMN) and MCL-AHN (or MCL-AMN)

SM-AHN is an entity comprising all cases of SM with an AHN [2,3].

Despite being rare, SM-AHN represents the second most frequent subtype after ISM, with a frequency between 21% and 44% [23,35].

A large German study by Horny et al. reported 20 cases of SM-AHN, among approximately 19.500 routinely processed BM biopsies evaluated in a reference center for hematopathology between 2000 and 2003 [23]. In this large study, ISM was diagnosed in 35 cases, ASM in 7 cases and MCL in only 2 cases.

As hematologic malignancies associated with SM are myeloid neoplasms in over 90% of cases, whereas lymphoid or plasma cells malignancies are rarely associated with SM and are clonally unrelated, in the 2022 ICC, the category of SM-AHN has been changed to SM-AMN (Table 4) [10].

The associated myeloid neoplasms often share *KIT* mutations and/or other genetic abnormalities with SM, whereas *KIT* mutations are not found in lymphoid or plasma cell neoplasms co-occurring with SM [10].

Myeloid malignancies associated with SM may be myelodysplastic neoplasms (MDS), myelodysplastic/myeloproliferative neoplasms (MDS/MPN), AML and chronic myeloproliferative neoplasms (MPN).

According to all the published data, chronic myelomonocytic leukemia (CMML), which is included in the group of MDS/MPN, is the most frequent myeloid neoplasm associated with SM [2,23,36].

All subtypes of AML may occur in association with SM, with a prevalence for AML types M2 and M5 [36].

The association of SM with lymphoid or plasma cell neoplasms is rare (10% of cases) and, interestingly, monoclonal gammopathy of undetermined significance (MGUS) is more frequent in SM patients than overt plasma cell myeloma or lymphomas [23,24].

The diagnosis of SM-AHN (SM-AMN) represents a diagnostic challenge for pathologists, and it is based on a thorough evaluation of BM biopsies with the aid of appropriate immunostainings to identify MCs.

In particular, a diffuse blast proliferation of AML may obscure a coexistent neoplastic MC infiltrate, which may be easily overlooked if appropriate immunohistochemistry is not performed. A second BM performed after induction chemotherapy for AML may reveal compact MC infiltrates which become evident due to the pronounced therapy-related aplasia of myeloid blast cells [37,38]. AML with t(8;21) is frequently reported to be associated with SM, and MC neoplastic aggregates may be overlooked in the initial BM biopsy [23,37,38].

According to Horny et al., the incidence of SM-AHN (SM-AMN) would increase if appropriate immunostainings such as tryptase, CD117 and CD25 were applied routinely in all BM biopsies containing infiltrates of MDS, AML, CMML and MPN [23].

It has been supposed that both SM and the associated hematological malignancy may originate from an early uncommitted hematopoietic cell with a subsequent progression into phenotypically different clones. This hypothesis is considered the most likely in cases of SM associated with myeloid malignancies, whereas the hypothesis of the coincidental occurrence of two different clonal hematological neoplasms may be more likely for the rare event of SM associated with lymphoid or plasma cell malignancies [10,23,37,38].

The detection of SM associated with another hematological malignancy has important prognostic and therapeutic implications, as MC neoplastic proliferations are often resistant to the majority of cytoreductive drugs employed in myeloid neoplasms. Detecting both neoplasms is crucial to offer therapies that could target both diseases.

In the 2022 MCL study by Kennedy et al., approximately 34% of patients had a diagnosis of MCL-AHN [9].

The most common AHN was myelodysplastic syndrome/myeloproliferative neoplasm unclassifiable (MDS/MPN-U; 45.2%), followed by CMML (25.8%), chronic eosinophilic leukemia (CEL; 9.7%) and AML (9.7%), whereas non-Hodgkin lymphoma and plasma cell myeloma were found in 3.2% of cases [9].

## 10. Differential Diagnosis between MCL and Myelomastocytic leukemia (MML) and Other Rare Entities

MML is a rare but probably understated type of leukemia characterized by an increase in immature MCs and metachromatic blasts in the context of an advanced myeloid neoplasm (usually AML, MDS with excess of blasts or an accelerated/blast phase of either MPN or MDS/MPN).

If tryptase and CD117 were routinely applied in the diagnosis of all cases of advanced myeloid neoplasms, it is estimated that MML would be identified in 3% of myeloid neoplasms [11,39,40].

By definition, SM diagnostic criteria are not present in MML; in particular, the compact aggregates of SM are not identified in MML, CD25 is negative, and KIT mutations are absent.

The diagnostic criteria of MML are the following:

- at least 10% immature MCs/metachromatic blasts in BM smear in the context of an advanced myeloid neoplasm;
- >5% myeloblasts in BM smear or PB (sign of advanced myeloid neoplasm);
- at least 10% of BM cells should be MCs (positivity for tryptase and CD117);
- absence of SM diagnostic criteria (in particular, MML is CD25-negative and lacks KIT mutations).

The distinction between MML and MCL may be difficult; however, the presence of immature MCs expressing CD25 and the identification of *KIT* mutations are in favor of MCL diagnosis [11,39,40]. The criteria for discriminating between MCL and MML are summarized in Table 5.

MML needs to be differentiated also from tryptase-positive AML [41].

This rare form of AML is characterized by an increased serum tryptase level similar to that of MCL and MML; however, in the majority of tryptase-positive AML, only tryptase and not CD117 is co-expressed by CD34-positive myeloblasts [41]. On the other hand, the co-expression of tryptase and CD117 by CD34-negative cells renders the diagnosis of MML more likely.

MML needs to be separated from SM/AHN, in particular when the AHN is represented by AML. In patients with SM/AHN, the diagnostic criteria for SM need to be present, whereas by definition, they are absent in MML.

Finally, both MCL and MML should be distinguished from acute basophilic leukemia and chronic basophilic leukemia [11,39,40]. Acute basophilic leukemia is characterized by the presence of metachromatic blasts often co-expressing tryptase and CD25, whereas strong expression of CD117 is not found; in MML, immature MCs co-express tryptase and CD117, but lack CD25 expression. Unlike in MCL, serum tryptase level is only slightly/moderately elevated, and no *KIT* mutations are found in MML and acute basophilic leukemia. The diagnosis of acute basophilic leukemia needs to be further confirmed by the use of immunohistochemical stains for basophil-specific antigens such as BB1 and 2D7.

Chronic basophilic leukemia is characterized by mature and often hypo-granulated basophils expressing tryptase, but not CD117. Blood basophilia is typically present in chronic basophilic leukemia and absent in MCL, acute basophilic leukemia and MML.

## 11. MCL: Outcome and Treatment

The prognosis of MCL is significantly worse than other subtypes of SM, with an OS ranging from 0–5 to 2.6 years, and no therapy is capable of inducing a durable long-term response, although encouraging results have been obtained with midostaurin [4,5,6,9].

In a study by Kennedy et al., MCL patients had an OS that was significantly inferior (1.6 years) to patients with ASM (6.2 years) and SM-AHN (2.8 years) [9].

The median OS of the entire large MCL cohort recently evaluated by Kennedy et al. was 1.6 years, although the following differences were present, depending on the MCL subtype [9].

In detail, patients with MCL-AHN had an inferior OS compared to patients with MCL alone (1.3 versus 2.3 years); patients with leukemic MCL had an OS inferior to patients with the aleukemic form (0.4 versus 1.9 years); and patients with any circulating MCs had an OS inferior to patients with no circulating MCs (0.5 versus 3.2 years).

No difference in survival outcomes was observed between patients with de novo MCL and patients with sMCL; by contrast, the presence versus absence of C-findings, which were used to define acute and chronic MCL, respectively, was highly significant, as acute MCL had an OS inferior to chronic MCL [9].

In addition, Kennedy et al. observed that *KIT D816V*-positive patients had superior OS compared to *KIT D816V*-negative patients (3.2 versus 0.9 years), and interestingly, patients with alternative *KIT* mutations had an OS similar to patients without *KIT* mutations.

As previously mentioned in the paragraph on MCL molecular features, it has been demonstrated that the presence of *S/A/R* mutations reduces OS in MCL patients [5,9,34].

In conclusion, the study by Kennedy et al., demonstrated that a diagnosis of MCL-AHN, the presence of any circulating MCs, abnormal karyotype, *KIT D816V* negativity, *S/A/R* mutations and treatment status (not receiving midostaurin) were all associated with inferior outcomes; whereas chronic MCL, *KIT D816V* positivity and midostaurin therapy at any point were associated with superior OS [9].

In the study of Jain et al. reporting a single-center American study, the OS was longer than in the German study by Jawhar et al. (31 versus 17 months) [5,6]. This difference in survival could be explained by a higher percentage of patients with AHN in the German study compared with the American study (70% versus 30%). Another explanation could be the higher number of *S/A/R*-positive cases observed in the German study compared with the American report [5,6].

For MCL, few therapeutic options are available, and due to the rarity of the disease, no approved standard therapy exists.

Cytoreductive chemotherapy has been used for MCL treatment; most frequently, single-agent 2-CdA or multi-agent AML-type induction regimens have been used [4].

A possible option for young and fit patients is represented by ASCT when a response to 2-CdA and/or chemotherapy is obtained [4]. ASCT needs to be performed early, as responses to 2-CdA or intensive chemotherapy are not durable, and in particular, AML-type induction chemotherapy seems to be of scarce efficacy in MCL [9].

However, because of advanced age, disease-related organ damage and poor response to chemotherapy, only a small proportion of patients is eligible for ASCT.

In addition, only rarely complete remission is obtained after transplantation. In a retrospective report, it was observed that among advanced SM undergoing ASCT, MCL patients showed the worst outcome, with a three-year OS of 17% [9,42].

Encouraging results have been obtained with KIT-targeting drugs, particularly midostaurin.

In 2017, midostaurin, a small-molecule inhibitor of multiple type III tyrosine kinase receptors, has been approved by the Food and Drug Administration (FDA) and by the European Medicines Agency (EMA) for patients with ASM, SM-AHD and MCL [6,9,43].

In the 2022 study by Kennedy et al., patients receiving midostaurin at any point during their treatment course had a superior median OS compared to patients non-treated with midostaurin (2.3 versus 1.1 years); the difference was even more significant for patients receiving midostaurin as a first line of treatment compared to patients who received a different therapy (3.2 versus 1.3 years) [9].

In 2021, avapritinib, a selective *KIT D816V* inhibitor, was approved by the FDA as a first-line treatment in patients with advanced SM and in 2022 by the EMA as second-line therapy [44,45]. The impact of this KIT-targeting agent on MCL prognosis requires further evaluation.

## 12. Conclusions

MCL is a rare and aggressive form of SM. The current available data on the disease are based on single reports, with only four large studies published so far. Despite its overall poor prognosis, some characteristics are relevant to predicting MCL patient survival. In particular, a diagnosis of MCL-AHN, the presence of *S/A/R* mutations and *KIT D816V* negativity are associated with a worse outcome. Midostaurin treatment at any point in the course of the disease is associated with a better prognosis. Further studies are essential to improve clinicians’ and pathologists’ awareness of the disease in order to provide an accurate and rapid diagnosis with consequent appropriate treatment.

## Figures and Tables

**Table 1:** Diagnostic criteria for MCL.

Criteria for SM required. In BM smear, at least 20% atypical immature MCs. ^1^

^1^ As per the 2022 ICC, if BM smear is suboptimal, a BM biopsy with diffuse infiltration of atypical immature MCs is accepted to establish MCL diagnosis.

**Table 2:** MCL variants.

Primary MCL (developing de novo) Secondary MCL (developing from a prior MC neoplasm)
Acute MCL (with organ damage, so-called C-findings and aggressive course) Chronic MCL (without C-findings and a more stable course, but evolving to acute MCL over a variable length of time)
MCL (alone) MCL with an associated hematological neoplasm (MCL-AHN) ^1^
MCL leukemic variant (with at least 10% atypical immature circulating MCs) ^2^ MCL aleukemic variant (with less than 10% atypical immature circulating MCs) ^2^

Legend: ^1^ MCL-ANH is renamed MCL with an associated myeloid neoplasm (MCL-AMN) in the 2022 ICC, as MCL is mainly associated with neoplasms of myeloid origin often clonally related to MCL; ^2^ in the 2022 ICC, it is recommended to report any circulating atypical immature MCs because of the negative prognostic significance.

**Table 3:** Diagnostic criteria for systemic mastocytosis.

2022 WHO: The diagnosis of SM can be performed when the **major criterion plus one minor criterion** or at least **3 minor criteria** are fulfilled. 2022 ICC: The diagnosis of SM can be performed when the **major criterion or, in its absence, at least 3 minor criteria** are fulfilled.
**Major criterion**
Multifocal, dense MCs aggregates (each aggregate = or >15 MCs) in BM biopsy and/or other extracutaneous organ
**Minor criteria**
>25% spindle-shaped or atypical or immature MCs in BM biopsy or BM smear or other extracutaneous organs Any kind of KIT mutation in BM, blood or other extracutaneous organ MCs expressing CD25 with or without CD2 (in addition to normal MC markers) MCs expressing CD30 Persistently elevated serum tryptase level (>20 ng/mL), unless there is an associated myeloid neoplasm, in which case, this parameter is not valid

**Table 4:** Hematological malignancies associated with SM according to 2022 WHO and 2022 ICC.

**2022 WHO: SM-AHN** AHN includes neoplasms of myeloid, plasma-cell and lymphoid-cell origin
**2022 ICC: SM-AMN** AMN includes only neoplasms of myeloid origin

Legends: SM-AHN: systemic mastocytosis with an associated hematological neoplasm; SM-AMN: systemic mastocytosis with an associated myeloid neoplasm.

**Table 5:** Diagnostic criteria of MML and MCL.

Myelomastocytic Leukemia	Mast Cell Leukemia
At least 10% immature mast cells or metachromatic blasts in BM	=/>20% immature mast cells or metachromatic blasts in BM
>5% myeloblasts in BM or PB	Immature mast cells may be present or not in PB
CD25-negative	CD25-positive
Wild-type *KIT*	*KIT* mutations in most cases

Legends: BM: bone marrow; PB: peripheral blood; MCL: mast cell leukemia; MML: myelomastocytic leukemia.

## Data Availability

The data presented in this study are available in this article.

## Abbreviation

AML: acute myeloid leukemia; ASM: aggressive systemic mastocytosis; BMM: bone marrow mastocytosis; CEL: chronic eosinophilic leukemia; CM: cutaneous mastocytosis; CMML: chronic myelomonocytic leukemia; ISM: indolent systemic mastocytosis; MCL: mast cell leukemia; MCL-AHN: mast cell leukemia with an associated hematological neoplasm; MCL-AMN: mast cell leukemia with an associated myeloid neoplasm; MCS: mast cell sarcoma; MDS: myelodysplastic neoplasm; MGUS: monoclonal gammopathy of undetermined significance; MM: multiple myeloma; MML: myelomastocytic leukemia; MPN: myeloproferative neoplasm; MDS/MPN-U: myelodysplastic/myeloproliferative neoplasm, unclassifiable; SM: systemic mastocytosis; SSM: smoldering systemic mastocytosis; WDSM: well-differentiated systemic mastocytosis.
